# Lancashire County Asylums

**Published:** 1902-02-01

**Authors:** 


### LANCASHIRE COUNTY ASYLUMS.

SINCE the publication of our former annotation on the Lancashire Asylums, we learn that Dr. Gemmel has been appointed Medical Superintendent of the Whittingham Asylum in place of Mr. Perceval, promoted to Prestwich. The appointment is one which will give general satisfaction in the specialty. Dr. Gemmel is a graduate of the University of Glasgow, and was for some time house physician in the Royal Infirmary of Glasgow. For several years he has been deputy medical superintendent of the Lancaster Moor Asylum where he has had the advantage of being under that able administrator, Dr. Cassidy.

Dr. Gemmel is author of one of the best monographs on that asylum scourge, ulcerative colitis, and has contributed some important papers to the medical journals.

